# Gender Differences in the Psychosocial Functioning of Parents During the COVID-19 Pandemic

**DOI:** 10.3389/fpsyg.2022.846238

**Published:** 2022-07-08

**Authors:** Katriona O’Sullivan, Nicole Rock, Lydia Burke, Neasa Boyle, Natasha Joksimovic, Holly Foley, Serena Clark

**Affiliations:** Department of Psychology, Assisting Living and Learning Institute (ALL), Maynooth University, Maynooth, Ireland

**Keywords:** gender difference, parental stress, COVID-19, family stress and coping, mother

## Abstract

The COVID-19 pandemic significantly affected family life, increasing parental stress around health, job losses, reduced salaries, and maintaining domestic life in lockdown and social isolation. The transition to home-schooling and remote work with school and workplace closures caused additional stressors as families began living, working, and educating in one place. This research aims to understand the relationship between the pandemic and parental stress, focusing on family well-being and established characteristics of the family unit that may cause some family members to experience the adverse consequences of the pandemic in more or less profound ways, especially mothers. Previous research shows that mothers carry more family responsibilities than fathers and can experience higher stress levels. This study employed a quantitative cross-sectional online survey to extend our understanding of the interaction between home-schooling, work and home life, and stress levels in a group of 364 parents. In total, 232 mothers and 132 fathers completed the survey. Results revealed mothers were 10 times more likely to be responsible for home-schooling than fathers, and 44% of mothers felt they had no help with home-schooling and were generally more stressed than fathers. These results show that lack of support, managing home-schooling, and being a mother predicted increased stress. 10% of mothers reported leaving their jobs due to pressure added by home-schooling. This study broadens the understanding of the pandemic’s impact on gender imbalances in family responsibilities. It emphasises the need for extra consideration for the impact on mothers as we emerge from this pandemic.

## Introduction

The COVID-19 pandemic significantly affected family life, increasing parental stress around health, job losses, reduced salaries, and maintaining domestic life in lockdown and social isolation. The transition to home-schooling and remote work with school and workplace closures caused additional stressors as families began living, working, and educating in one place (Westrupp et al., 2020; Clark et al., 2020; Riem et al., 2021). The effects of these challenges will likely be long-term partly because of how contextual risk becomes rooted in the structures and processes of family systems. How families handle and cope with pandemic challenges and the degree of impact these difficulties have on families will likely vary considerably. For some families, the consequences of the pandemic may cause increased psychological distress resulting in greater reliance on less effective parenting practices or behavioural changes in children. At the same time, other families may experience less significant impacts in managing and dealing with the pandemic (Riem et al., 2021).

The impacts of the pandemic on families’ well-being are only beginning to be understood. Despite this, one-third of families reported being ‘very or extremely’ anxious because of family stress resulting from pandemic related confinement (Prime et al., 2020). Prime et al. (2020) argue that studying pandemic related family stress and well-being is part of the social justice mandate of scientific and professional psychology to respond to the suffering of families and children. This helps them deal with hardships, particularly those underrepresented or experiencing marginalisation. Therefore, they argue it is essential for practitioners working with families and children to understand the consequences of the pandemic on these groups.

This article describes the relationship between the pandemic and parental stress, focusing on family well-being and established characteristics of the family unit that may cause some family members to experience the adverse consequences of the pandemic in more or less profound ways. Specifically, it examines the pandemic’s effect on families and parental stress relating to home-schooling and how the added responsibility of home-schooling impacts parents’ ability to work and parent concurrently and effectively. The following background, methodology, analysis and discussion are designed to add to the growing knowledge base on psychological health. Doing so offers essential findings to inform practitioners and the scientific and academic communities.

### COVID-19, the Family Unit and Parental Stress

The social and economic disruptions of COVID-19 are exacerbating parental stress relating to financial insecurity, caregiving burden, reduction in social support outside the family unit, changes to work routines, and the need to meet children’s social and educational needs. The pandemic is causing families to face unprecedented adversities, and with this dynamic, there is an essential need for strong family leadership supported by nurturance, guidance and protection. However, this authoritative parenting approach often breaks down in times of anxiety and uncertainty, increasing parental stress and straining the family unit. When parents face heightened stress levels, mental and emotional resources diminish, making positive family leadership more challenging and causing overreliance on less effective parenting methods.

The conceptual framework of this research draws on Prime et al. (2020), using systematic models of human development and family functioning to connect pandemic related social disruption to parental stress. To understand these correlations, the present study examines parental well-being and family processes, such as communication, beliefs, and organisation. Specifically, it looks at the relationship between parental stress, home-schooling and the ability to work and parent at the same time during COVID-19.

Prime, Wade and Browne developed this framework to link social disruption due to the pandemic to child adjustment using a ‘cascading process’ relating to the well-being of caregivers and family processes. Still, the framework can also adapt to address similar topics, such as the one in this study. In their framework, Prime, Wade and Browne combine family systems theory, the bioecological model, the family stress model and the developmental system theory. Combining these approaches, they show how pandemic-related disruptions generate higher levels of psychological distress for parents, influencing the quality of relationships between caregivers, parents and children and siblings. These changes in relational dynamics can disrupt children’s adjustment.

By drawing on these principles, this current study illustrates that contextual risks (home-schooling, remote work, family dynamics) and social disruption pose significant parental stress risks. The connections between adversity, parental stress and family well-being are not unidimensional. Instead, as Prime, Wade and Browne demonstrate, they operate in a reinforcing system where stress and disruption in one domain cause stress and disruption in another. Existing vulnerabilities in families may exacerbate social disruption and prolong the consequential outcomes of the pandemic. For example, stressors that hinder the functioning of one family member can cause changes to how other family members function. At the same time, reduced parental stress and increased parental and family well-being can safeguard against these stressors (Prime et al., 2020).

### COVID-19, Gender Dynamics in the Family Unit and Parental Stress

The pandemic made it necessary for parents to be full-time caretakers and teachers without the ability to rely on help outside the immediate family unit (Pew Research Center, 2015). At the same time, many parents had to maintain paid work, and others had to cope with the consequences of unemployment due to COVID-19. Meanwhile, essential workers had the added stress of finding childcare and the risk of exposing their families to COVID-19. These dynamics altered many dual-earning couples’ domestic labour divisions, forcing them to manage a reorganisation of childcare, work, and home-schooling.

Before the global pandemic, mothers in heterosexual relationships did most family-related work, even as breadwinners. In a culture that encourages intensive mothering ideology, COVID-19 exacerbated gender inequality, with home and childcare responsibilities falling more on women. Intensive mothering is an ideology developed from traditional gender norms that impose unrealistic motherhood standards to determine the status of a ‘good mother’. This ideology holds that mothers are the ‘best’ and ‘preferred’ caregivers and must care for their children’s health and development needs. From this perspective, mothering should be ‘child-centred, expert-guided, emotionally absorbing, labour-intensive, and financially expensive’ (Hays, 1996).

Intensive mothering can decrease mothers’ mental health and establish an unequal division of labour. Though this philosophy focuses on mothers, it creates a ‘complementary social script for fathers’ (Hays, 1996). Even if parents have similar parenting attitudes, gender norms pressure women to take on most domestic duties. Further to this, research suggests that women and men understand the meaning of being a ‘good’ parent differently. Men generally define a good parent by the ability to provide financial stability for the family. In contrast, women must often put their needs second to the responsibilities of motherhood, including personal needs, well-being and career (Hays, 1996). These gender dynamics may influence differences in parental stress levels for fathers and mothers, especially during times of crisis, such as the COVID-19 pandemic. Moreover, social theory highlights women’s challenges when balancing mothering roles versus work life. Mothers tend to take more responsibility for caregiving and domestic duties than fathers. While men acknowledge the importance of domestic responsibilities, they are prone to ignore them (Thébaud et al., 2019), a trend amplified during COVID-19 (Alon et al., 2020). Baumeister and Leary (1995) find that conforming to social norms is essential for people to feel that they belong. With is, people are motivated to adhere to social norms as they are rewarded when they do and punished when they deviate from them (Baumeister and Leary, 1995). In society, motherhood is idealised as the ‘supreme physical and emotional achievement in women’s lives’ (Phoenix et al., 1991). Being able to meet the expectations and fulfil the ‘norms’ of motherhood is considered significant for affirming a mother’s social identity and her secure sense of self (Gaunt, 2008). To meet these standards and affirm their identity as a mother, women often take ownership of household and childcare duties, carrying out these tasks themselves and setting the standards for how they need to be completed in the home. This phenomenon is known as maternal gatekeeping and is motivated by a fear that they will be socially judged for their housekeeping and childcare abilities if they do not meet the social standard (Gaunt, 2008; Puhlman and Pasley, 2013). Studies indicate that these pressures on mothers are related to higher levels of maternal guilt, burnout, and increased stress levels (Henderson et al., 2016; Borelli et al., 2017; Meeussen and Van Laar, 2018). Research shows that guilt, shame and stress generally involve a social evaluation piece and the fear of being judged within society for failing to reach mothering standards (Gilbert, 1998, 2007; Deonna and Teroni, 2008).

According to human capital theories (Becker, 1985), unequal responsibilities for domestic life have a broader impact than just increasing mothers’ psychological stress; they also interfere with a mother’s ability to stay engaged in the labour market. Before COVID-19, reduced involvement in domestic tasks enabled fathers to be more committed to the workplace; maternal shame and the cultural expectations of intensive mothering forced women to prioritise domestic tasks, creating disadvantages for women in the labour market (Benard and Correll, 2010). This is seen in fathers spending longer hours at work than mothers and mothers being more likely to reduce their work hours or even quit jobs if work interferes with their family responsibilities (Kaufman and Grönlund, 2019). Consistent with social theories which highlight intensive mothering expectations, mothers are more pressured to miss work when there are problems with childcare or schooling (Maume, 2008). COVID-19 has increased pressure on families; more specifically, home-schooling has added to the pressure felt by working mothers (Clark et al., 2020). Evidence is emerging that guilt and stress are increasing for mothers (Brown et al., 2020). The changing pressures being placed on families during COVID-19 may also be increasing the risk of depression and anxiety among parents. There are growing indications that it is having a negative impact on the psychosocial functioning of families (Clark et al., 2020; Westrupp et al., 2020; O’Sullivan et al., 2021). A study by Malkawi et al. (2020) indicated increased depression, anxiety, and stress levels in mothers during the COVID-19 pandemic compared to fathers. These findings align with previous research that highlights females are more prone to experience depression and anxiety (American Psychiatric Association, 2013), especially in times of stress (Momayyezi et al., 2020). A lack of social support, having children with additional needs and having a more significant number of children and conflict within relationships can increase the risk for maternal depression (Wachs et al., 2009; Rahman et al., 2013; Gelaye et al., 2016). Increased social support and proactive outreach could enhance prevention and more rapid treatment (Almeida et al., 2020). Research shows that the parenting participation of fathers can moderate maternal stress. Fathers can play a protective role, negotiating the impact of maternal stress by reducing the damaging effects of maternal parenting behaviour on family well-being. This has been observed in stressful situations that increase the risk of maternal distress, such as COVID-19 (Papadaki and Giovazolias, 2015).

In 2020 Clark et al. interviewed families, exploring their experiences of COVID-19 pandemic restrictions. Results revealed that working mothers struggled to manage the added burden of home-schooling. There was evidence of increased stress and isolation; mothers were particularly concerned about the longer-term impact of the pandemic on their families and their ability to participate in the labour market. This research extends these findings by collecting survey-based data to understand parental stress during Ireland’s second wave of COVID-19 restrictions. The survey aims to understand parental experiences of home-schooling and working (or not) during the pandemic and how these experiences correlate with stress.

The paper tests specific research questions based on the research literature and the study conducted in 2020 by Clark et al. These include:

1. Did parents who worked full-time during the COVID-19 pandemic experience higher levels of stress than parents who worked part-time or who did not work?
2. Did parents who worked full-time during the COVID-19 pandemic find home-schooling harder than parents who worked part-time or who did not work?
3. Does family structure, number of children, home-schooling, and parent type predict parental stress during COVID-19?
4. Did mothers and father differ in their experiences of home-schooling and work, their stress during COVID-19, and their perception of support?
5. What are the experiences of families during COVID-19?

## Materials and Methods

### Design

This study employed a quantitative cross-sectional online survey design to assess the relationships and associations between variables. This design also featured the inclusion of qualitative questions regarding the participant’s experiences of home-schooling. An Interpretative Phenomenological Analysis (IPA) approach (Smith and Shinebourne, 2012) was used to analyse the qualitative data obtained in this study.

The survey’s methodological design was integral because the qualitative questions provided the researchers with a more in-depth understanding to support the quantitative data further. The survey enabled participants to expand their responses to the quantitative questions by including opportunities for open text answers. The information collected in the qualitative questions added valuable insights to the quantitative data.

### Participants

Four hundred and thirty-eight parents completed the online survey. Participants were recruited *via* social media and other networking platforms using snowballing sampling techniques. The data of 334 participants was fully complete and included in the final analysis. Of these 364 participants, 232 were female, and 132 were male. Inclusion criteria for the study were being a mother or father of school-age children and having at least some experience of home-schooling.

### Measures

The survey was designed to explore specific areas of the research questions. The sample’s demographics focused on several variables, including the number of children being home-schooled, which parent oversaw home-schooling, and family structure (e.g., lone parenting, lone parenting—with family support, co-parenting -both parents living together etc.).

The data collection focused on four areas: home-schooling, education structures and supports, stress, and work–life balance. Several questions accompanied each of these categories. For example, to measure the experiences of home-schooling parents, the survey sought to understand who has the primary responsibility for home-schooling in the home; confidence in home-schooling, confidence in partners’ abilities to home-school and help from partners in home-schooling. Regarding education and structures, survey questions concentrated on school supports, online schooling and satisfaction with education moving online. In measuring stress, questions focused on stress levels, time constraints (or not), feelings of letting down their family and guilt. Finally, to explore work–life balance, the survey accounted for hours worked, at home or in office work dynamics, and the impacts of home-schooling on professional life.

### Procedure

This study received ethical approval from The Social Research Ethics Subcommittee, Maynooth University (2407411). This study was completed using the Microsoft Forms platform. Before filling out the survey, participants were provided with an information sheet and a consent form before participating in the study. All participants were provided clear and concise information about the nature of the research and what the questionnaire would entail. They were informed that all the information they provided would remain anonymous and nonidentifiable and were reassured of their right not to participate.

Once this was completed, participants could begin filling out the questionnaire. Upon completion, a de-briefing sheet was provided to participants. Once all the data was gathered, it was coded to create a singular SPSS data file for analysis. Responses to the qualitative questions in the survey were analysed separately. These data were stored on a password-protected computer.

### Data Analysis

A power analysis was run before the conduction of this study and found that for this test to have sufficient power, 139 participants would be required. This was based on *a priori* sample size calculation assuming a medium effect size, a power of 0.8, 15 predictor variables, and a probability level of 0.05. All statistical analysis was conducted using SPSS version 26. Independent samples *t*-tests, one-way ANOVA, and binomial logistical regression analysis were run to investigate the relationships and associations between variables.

A thematic approach to IPA was used to analyse the qualitative data collected as part of this study. The analysis was guided by the six steps outlined by Smith and Shinebourne (2012):

1. Reading/Re-reading—The research team familiarise themselves with the qualitative data.
2. Coding—Codes are identified and organised into initial themes.
3. Clustering—Common themes and sub-themes are linked together to form the themes.
4. Iteration—The iterative process involves several revisions, including checking themes, sub-themes, and quotes.
5. Narration—A narrative was developed based on the findings. The narration process involves describing the themes and using quotes to illustrate them.
6. Contextualisation—The findings are interpreted within the context of existing literature.

## Results

### Descriptive Statistics

Table 1 compares the percentage of mothers and fathers who agreed with the following questions. The comparison shows that mothers are 10 times more likely to manage the home-schooling than fathers, and 44% of mothers feel they have no help with home-schooling compared to 17% of fathers. More fathers work full time, and more mothers work part time; however, 18% more mothers work from home. Mothers are three times more likely to report feeling pressure to leave work than fathers due to home-schooling responsibilities. Mothers are more stressed overall and feel like they are letting their families down at higher rates than fathers. 44% of mothers report that their career suffers due to home-schooling compared to only 18% of men.

**Table 1:** A comparison of the percentage of mothers and fathers who agreed with questions.

Category	Question	% Mothers who said yes	% Fathers who said yes
Home-schooling	I have primary responsibility for home-schooling	65	6
	I am confident in my ability to home-school	69	83
	I am confident in my partners’ ability to home-school	63	89
	I feel like I have no help home-schooling my children	44	17
Work–life	I work full-time	55	86
	I work part-time	31	1
	I work from home	88	70
	I feel pressure to leave work due to family responsibilities	22	8
	I can easily fit home-schooling around my work	8	44
	I have left my job due to home-schooling	10	2
	I have a good work family-work life balance	11	50
	My career is suffering due to home-schooling	44	18
	I am not working to my full potential due to home-schooling	71	41
Stress	I feel stressed overall	77	56
	I feel guilty about home-schooling	81	78
	I feel like I am letting my family down	57	25
	I feel like I do not have enough time enough time	91	76

### Impact of Work Type on Stress and Home Schooling

**Figure 1:**
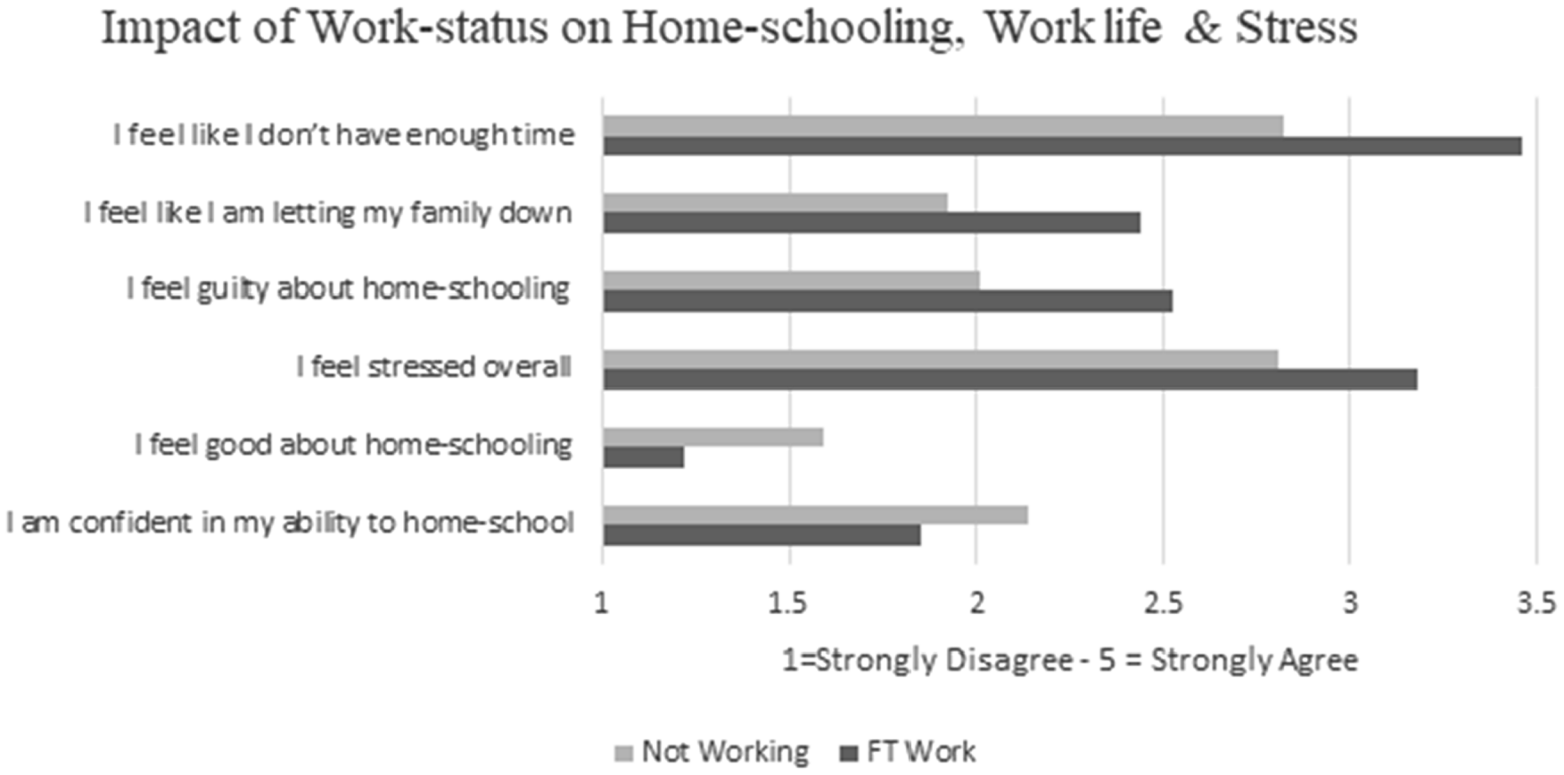
Average rating differences for questions relating to home-schooling and stress for full-time parents and parents not working.

To examine research question one, the study looked specifically at how being full-time or part-time would impact stress and home-schooling. To examine this, a series of independent sample *T*-Tests were performed (Table 2). The results show that parents who were not working full time were more confident to home-school and felt better about home-schooling than parents working full-time. Working parents were more stressed overall, reported not having enough time and felt they were letting their families down more than nonworking parents. Nonworking parents reported a better work–life balance than full time working parents (Figure 1).

**Table 2:** Means for the different family sizes for each question with significance levels of the one-way ANOVA.

Category	Question	FT work	Not working	*t*-value	*p*-value
Home-school	Confidence to home-school	1.85	2.14	−2.12	0.035
	Feel good about home-school	1.22	1.59	−2.41	0.017
Stress	Stressed overall	3.18	2.81	2.73	0.008
	Feel guilty about home-schooling	2.53	2.01	2.44	0.015
	Letting my family down	2.44	1.92	3.24	0.001
	Have enough time	3.46	2.82	4.74	0.001

### Factors Which Predict Parental Stress

Multiple linear regression analysis was used to develop a model for predicting parent stress score from family size, parent type (mother or father), working from home, working full time, managing home-schooling, guilt around home-schooling, work–family life balance, and help with home-schooling. Regression coefficients are shown in Table 3. Being a mother, working from home, working full time, managing home-schooling, having a good family–work life balance, and feeling like you had no one to help with homes-schooling predictors had significant (*p* < 0.05) partial effects in the full model. The eight-predictor model was able to account for 42% of the variance in parent stress, *F*(8, 308) = 29.03, *p* < 0.001, *R*^2^ = 0.42, 90% CI [−0.56, 0.65].

**Table 3:** Summary of linear regression analyses for variables predicting parent stress.

Variable	*B*	SE *B*	*t*	*p*
Number of children	−0.08	0.06	−1.26	0.21
Parent type	−0.29	0.14	−2.06	0.04
Working from home	0.43	0.11	3.78	0.001
Work Full time	−0.25	0.11	−2.16	0.03
I can easily fit home-schooling around my work	−0.17	0.05	−3.54	0.001
I feel guilty about home-schooling	0.04	0.03	1.10	0.27
I have a good family-work life balance	−0.29	0.05	−5.70	0.001
I feel like I have no-one to help me homes-school my child (ren)	0.19	0.04	4.56	0.001
Constant	3.25	0.29	11.16	0.001

### Different Experiences of Parents

**Figure 2:**
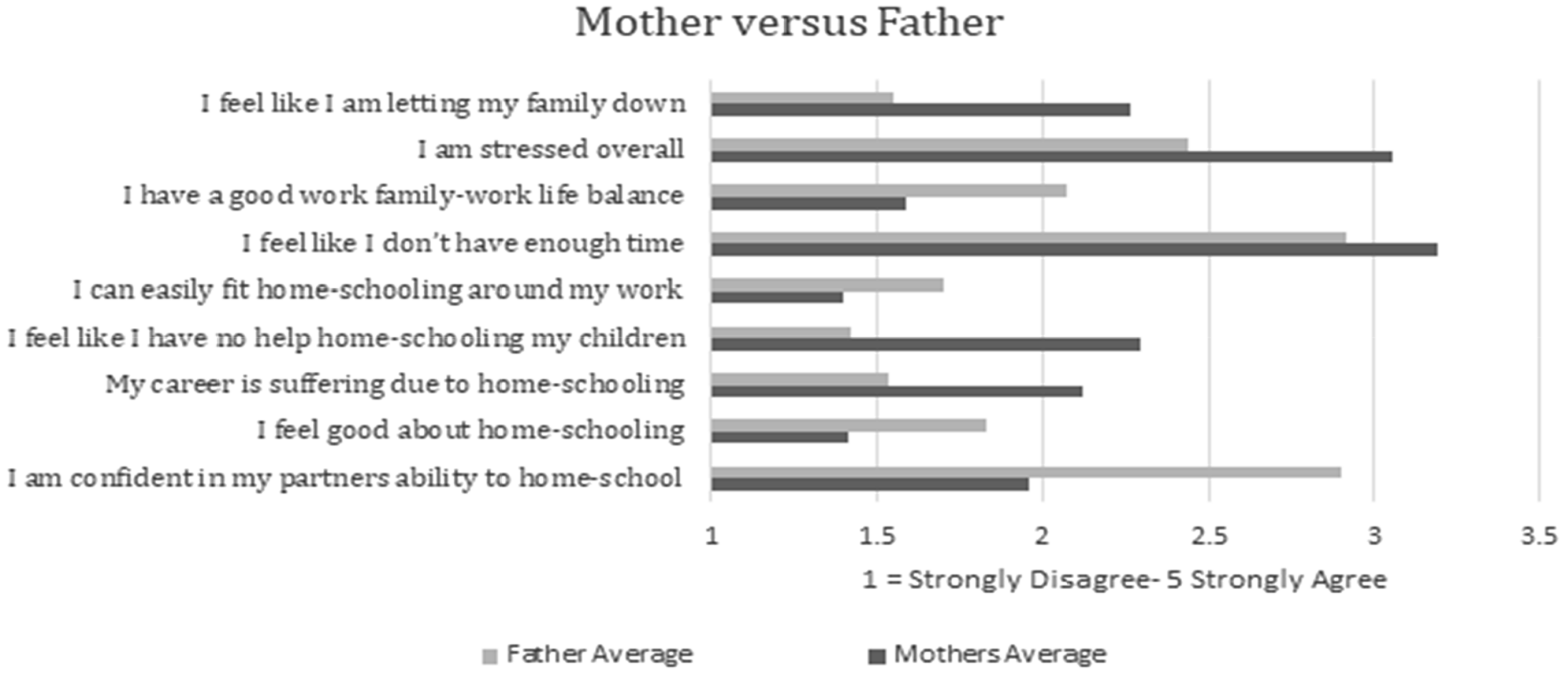
Average ratings differences for questions relating to home-schooling, work–life, and stress for mothers and fathers.

Independent Sample *T*-Tests were performed on several key questions relating to home-schooling, stress and work–life to establish if differences emerged for mothers and fathers. Table 4 reports the significant results; fathers feel better about home-schooling than mothers. They report having a better family–work life balance than mothers. They report being able to fit home-schooling around their work better than mothers. Conversely, mothers report their career suffering at higher levels than fathers. They feel like they have less help with home-schooling than fathers, they report higher levels of stress overall, and they report not having enough time at higher levels than fathers do (Figure 2).

**Table 4:** Independent t-tests mother versus father comparisons.

Variable	Mothers average	Father average	*t*	*p*
I am confident in my partners ability to home-school	1.96	2.9	−7.53	0.001
I feel good about home-schooling	1.41	1.83	6.43	0.001
My career is suffering due to home-schooling	2.12	1.53	−3.24	0.022
I feel like I have no help home-schooling my children	2.29	1.42	−3.76	0.001
I can easily fit home-schooling around my work	1.4	1.7	4.70	0.001
I feel like I do not have enough time	3.19	2.92	11.16	0.001
I have a good work family-work life balance	1.59	2.07	5.35	0.001
I am stressed overall	3.05	2.44	2.25	0.025
I feel like I am letting my family down	2.26	1.55	−3.34	0.001

## Qualitative Analysis

Three key themes were identified in the qualitative analysis, including concern about the impact of home-schooling on their children, stress, and pressure due to working from home. Each theme interrelates and elaborates on the above data.

### Theme 1: Concern About the Impact of Home-Schooling on Their Children

Parents expressed concern about home-schooling’s immediate and long-term impact on their children. In this regard, three sub-themes were identified: ‘Mental health difficulties’, ‘Getting on with it’, and ‘The schools have been unhelpful’.

#### Mental Health Difficulties

Respondents are worried about the impact that home-schooling and the lockdown is having on their children’s mental health; this is illustrated in the following responses:

*“I help where I can or when needed by my children. Their mental health is suffering more than their education as not seeing friends, no proper routine and all sports are off.”*

*“A nightmare … Younger children don't sleep because they are not active during the day… Supporting older children is very tough because they are under huge mental health strain…”*

*“My children are suffering as a result of being denied their right to an education. Online learning is an absolute farce, with classes being cancelled, pre-recorded etc. Basically, teaching themselves… They will regress in both schoolwork and sports and socialisation. They miss their friends and are down. Not their normal happy selves at aged 14…”*

Parents repeatedly describe highly stressed environments that are having a negative impact on their children. Particularly evident was the emotional strain on both parents and children. Mother’s reporting crying most days, alongside their children.

*“Terrible during spring lockdown. Tears every day, mine and/or theirs… As well as working part-time, I'm also a full-time student with classes on Zoom, with mandatory attendance. First day today was so busy, starting work shift at 6.30am for 3.5 hours followed by 4.5 hours of college. Ended up snoozing on sofa at 6.30. Will try harder tomorrow…”*

*“Extremely stressful trying to assist my son who has special needs and is suffering from extreme anxiety due to the pandemic and we're living alone.”*

The concern about mental health consequences stems from several challenges that parents identify as being particularly stressful for their children: the loss of friendships, the lack of sport and activity and the change in routing, including late sleeping. These challenges all merge into a picture of failing families and children missing out on a normal childhood.

#### Getting on With It

Many parents expressed the view that despite the challenges that home-schooling, and the lockdown were presenting them with, they were ‘getting on with it’.

*“We do our best, I don't pressure the kids, they attend every class, and complete all work assigned, it's not as good as school, they would be learning more and benefiting from social interaction, but these are not ordinary times, it will pass”*

*“I don’t believe we are home schooling, I am not planning lessons, setting targets etc. We are doing schoolwork at home, the teachers do the heavy lifting. I am not pressuring my children to achieve. We are focusing on good mental health, exercise, creative and fun activities”*

*“Many positives for my children and my relationship with them. Many challenges also… we have managed to work through them together and accepting that the best we can do is ‘good enough’. …government was not going to… provide the necessary supports. That was possibly the most challenging thing to accept…”*

The ability of some parents to persevere in challenging times is evident here. Those parents have to work extremely hard to protect their children from the consequences of the pandemic is also apparent.

#### The Schools Have Been Unhelpful

Many of the respondents expressed the view that the education being provided by their children’s schools is inadequate. However, this was not seen across all respondents, and there were clear differences between how families viewed the school’s supports. In terms of the people who were afraid of the impact of future pandemic restrictions, we see that they point to a lack of school support as being part of that fear. For example, parents said:

*“If support from school had been better with some live classes and supports, I feel it would have greatly increased the learning & happiness of the children. Work has never been busier, I find by the time the kids get up at 9/10 I have 3- or 4-hours work done, it's tough…”*

*“Awful, kids are upset and miss school. Teachers are not supportive. No interaction with teachers. No zoom classes. Don't get calls back from teachers. Told we can't discuss distance learning at parent teacher meetings. The children have been let down terribly.”*

This theme emerged in different ways whereby parents were comparing their effort with that of the schools, and some parents point out that they are doing their best to home-school their children and work from home. They do not feel like the schools are matching their efforts

*“Miserable. Minimum support from school. One Zoom class a week for a 5- and 8- year-old of half an hour duration. Meanwhile I am expected to deliver a full curriculum. Fails utterly to acknowledge the reality of life in lockdown for working parents”*

*“Horrific. I am a frontline worker so therefore am not at home to supervise home-school. The dept of education and teachers of Ireland are codding themselves if they think that stressed out working parents can educate the children of Ireland in the same way school does”*

While there are parents concerned with the schools’ level of support, there were times when parents were positive and happy with the support they received from their children’s school. However, references made to school support were generally negative and reflected the stress and concern parents have for the longer-term impact of the pandemic.

Among the families who are positive about their children’s schooling, some indicate that the level of stress from work and family life is too much to bear,

*“The school can't do anymore they are fantastic, but I am exhausted, and my kids can do a lot on their own, I feel low level depressed, I wish my organisation provided more support other than links to websites for help or the eap programme, considering going sick for a few weeks.”*

### Theme 2: Stress

The theme that came up repeatedly across the responses was that parents and children are under considerable stress. Of significance here is that even among those reporting positively, they too report being under stress. Respondents frequently described their family environments as difficult for everyone:

*“It is stressful due to the different ages. The youngest is still in primary school which has its own challenges. However, having 3 teenagers all having to be online at once in separate areas of the house has not been easy”*

*“Home-schooling one child with very complex needs demand fulltime care but I need to share that time with his sibling. He is struggling to cope with not having any social interaction or having anything to look forward to”*

Throughout the responses, there are curt replies that are indicative of the strain that parents are under. One-word and two-word replies are common, ‘Stressful’, ‘Highly-Stressful’, and those indicating parents are having a tough time.

*“Nightmare when I do it because I’m dyslexic.”*

*“It is one thing I would not like to do in the future as it can be very stressful.”*

Even among families that expressed the view that home-schooling and the lockdown have been a positive experience, some indicate that they too are finding it stressful:

*“Nice to have them at home when young, learning things myself, enjoying baking, and practical but very stressful with no break from each other at home.*”

*“Pressure!!!Overwhelming!!! I actually feel sad and I thankfully don't feel like that until the dreaded school closure announcement.”*

It is clear from the above responses that the pandemic, the lockdown, and the demands of home-schooling, are causing tremendous stress all through society and children are not immune from this.

### Theme 3: Pressure Due to Working From Home

The survey data reveal that parents working from home while home-schooling their children are finding the situation difficult. They are likely to express guilt about their inability to carry out their job and home-school their children effectively. There is also evidence that they are coming under increased pressure from unsympathetic employers. This can be seen in these responses:

*“Awful. My daughter misses the social interaction, and it is hard to keep her motivation up. Her school has said interaction with teachers is discretionary and based on teacher's capacity. My job expects me to perform regardless of my capacity and I am struggling to teach and work and do everything alone.”*

*“Would be completely fine if I wasn't working. As it is, it's a nightmare! My employers make no concessions and my daughter just doesn't get the attention or stimulation she needs.”*

*“I can home-school or I can do a good job in my work but not both. Home-schooling has been a challenge the children are losing interest I lack the time to motivate them”*

That parents are feeling a deep sense of guilt is also apparent:

*“Dreadful. It is just a nightmare while trying to work. The mummies who don't work love it and are sending in heaps of work and WhatsApp messages. I don't know any working mum who doesn't absolutely hate and resent the ridiculous bullshit situation we are in. It is completely nuts and must stop.”*

*“When I spend time it works well. Juggling work and trying to give time to home-schooling is stressful. I need to structure my day better”*

*“Home-schooling a special needs child who is struggling to manage his anxiety due to the pandemic as well as working from home and studying myself is extremely difficult I unfortunately can't give [100%] to everything which is frustrating as a parent”*

These responses indicate that parents working from home are putting themselves under pressure and are being put under pressure by their employers. There is pressure to fill the gap in their children’s lives that the absence of school has left, and they are coming under real pressure to prioritise their paid employment at the expense of time spent with their children. This attitude from employers calls into question the notion that the pandemic has had a unifying effect on society. Shouts of ‘we are all in this together’ ring hollow parents who must deal with a suffering child while your boss threatens to fire them for not giving him a solid eight-hour day from nine to five.

## Discussion

This study explored parental stress during the COVID-19 pandemic relating to home-schooling, traditional gender norms, labour divisions in the family unit, and work–life balance. It broadens the knowledge base on the impact of COVID-19 on parental stress and family well-being, fulfilling the social justice mandate of scientific and professional psychology to respond to the suffering of families and children, as discussed by Prime et al. (2020). It also offers essential information for practitioners and scholars working with families and children. It is particularly relevant for those working in the Irish context because, as Guo et al. (2020) found, mental health problems related to parental stress due to COVID-19 are not identical across country contexts. This study aligns with past research showing the close relationship between gender roles, divisions of labour, work–life balance and parental stress during COVID-19 (Thébaud et al., 2019; Clark et al., 2020; Guo et al., 2020; Prime et al., 2020; O’Sullivan et al., 2021; Riem et al., 2021; Forbes et al., 2022). These studies emphasise the causal relationship between these factors and increased maternal stress due to higher degrees of family-life responsibilities than fathers.

In the present study, mothers reported being the primary caregivers while also managing home-schooling, a trend amplified during COVID-19 (Alon et al., 2020). The initial examination of the distribution of the burden of home-schooling shows that mothers are taking primary responsibility for this, and working parents, especially mothers, have more negative home-schooling experiences than those who do not work.

One element of this study examines if there is a connection between labour market engagement, home-schooling due to COVID-19, and stress-related outcomes due to this dynamic. The data suggest that working parents had higher stress levels, more time constraints, and stronger feelings around disappointing their families than nonworking parents. Nonworking parents experienced less stress and a better overall life balance than full-time working parents and felt more equipped to and better about home-schooling their children. These observations add to the research showing that COVID-19 has increased pressure on families, particularly those with parents working full-time and managing home-schooling (Clark et al., 2020).

These results also support evidence demonstrating that COVID-19 impacts the psychosocial functioning of families, especially among those with full-time working parents (Clark et al., 2020; Westrupp et al., 2020; O’Sullivan et al., 2021). This observation is magnified across the sexes when considering working mothers versus working fathers. The data in this study illustrates that fathers feel better about home-schooling than mothers. They reported having a better family–work life balance and a better ability to structure home-schooling around their work schedules. Conversely, mothers reported their careers are suffering at higher levels than fathers. They feel like they have less help with home-schooling than fathers and have higher parental stress levels overall.

One essential factor that may mitigate the impact of COVID-19 on maternal caregiving is allomaternal care. This type of care refers to childminding by adults other than the mother, including grandparents and fathers. Evidence from research with high-risk families demonstrates the importance of allomaternal support. For instance, long-term adverse effects on maternal depression during a child’s infancy diminish when there is father support, suggesting that father involvement may reduce maternal stress. Conversely, if there is low or no father involvement, the risk of mothers abusing or neglecting their children increases. This finding is critical because the reported lower father involvement in labour division in family units, including childcare and home-schooling during the pandemic, could be a predictive factor in the likely long-term effects of the pandemic.

Building on this point, mothers also report higher levels of time constraints than fathers, which aligns with the work of Malkawi et al. (2020), observing increased stress in mothers during the COVID-19 pandemic compared to fathers. Intensive mothering and social theory highlight women’s challenges when balancing mothering and work–life (Baumeister and Leary, 1995). The ability to meet the expectations and fulfil the “norms” of motherhood is considered significant for affirming a mother’s social identity (Gaunt, 2008). This study suggests that working mothers feel differently about their home-schooling responsibilities than fathers, and they are more stressed and less satisfied. Fathers report more time for themselves. While they are aware of the burden being placed on mothers, the qualitative research shows that they are leaving the responsibility to the mothers rather than easing the load. It also points to a new shift in gender-parent inequality where mothers are expected to work and manage the “second” shift of housework and must also manage the home-schooling and the worry about the pandemic restrictions mean for their children.

In national and political responses to the pandemic, the potential cost that maternal stress has on family dynamics (Gilbert, 1998, 2007; Deonna and Teroni, 2008) is not being discussed. The longer-term negative consequences occurring developmentally for children living through this period are being ignored. The qualitative research from this study and that of Clark et al. (2020), shows that parents are aware of the pandemic’s impact and are stressed about what they perceive as its long-term implications for their children. This research shows how families feel isolated and unsupported by their schools and at times unable to cope with the new normal that COVID-19 presents them with. Without support, families will be left to recover from a complex mixture of fear, stress, and extra work—without any clear plan from those who are formulating policy responses to the crisis.

When accounting for the factors which predict parental stress, it is evident that being a mother was a significant indicator of stress while working from home, working full time, and managing home-schooling also predicted stress levels. Those who had a good family–work life balance, most of which were fathers, had less stress. Importantly, feeling like there was no one to help with home-schooling was a predictor of parental stress. This shows the significant impact of home-schooling on parental stress and how mothers are most at risk of having increased stress levels than fathers. Malkawi et al. (2020) observed increased stress in mothers during the COVID-19 pandemic but could not quantify the factors that contributed to this stress. Our research shows that home-schooling, and having no help were essential contributors to parental stress. When understanding how we emerge from the pandemic and how we shift back to some form of normality, the cost of the crisis and extra burden on mothers must be considered. Being asked to manage home-schooling and the responsibility of the second shift saw 10% of the mothers in our sample leave their jobs.

In comparison, only 2% of the fathers left their jobs due to the demands of home-schooling, meaning that 23 of the working mothers in our sample left work due to the added burden that home-schooling bought into their lives. This represents a substantial number of women who felt pressured to leave their jobs; we also see that 44% of working mothers said that their careers were suffering due to the added pressure of home-schooling. This finding points to the difficult choices facing many mothers when considering the balance between home and work. In many cases, some families reported stress and fear. The qualitative themes were stark. Parents repeatedly paint pictures of highly stressed environments that harm their children alongside mothers reporting crying most days with their children. This study expands on the research carried out by Clark et al. (2020), showing a correlation between parental stress, home-schooling and family well-being. It further broadens the understanding of the impact of COVID-19, revealing that mothers are bearing the brunt of the home-schooling responsibilities and are experiencing higher stress levels than fathers. The results show significant factors contributing to parental stress that need to be considered as the world emerges from the pandemic. These include family size, availability of support, and work–life factors. We have observed that mothers take primary responsibility for the home-schooling. They have experienced higher stress levels than fathers, and this stress can potentially impact the whole family negatively.

## Data Availability Statement

The raw data supporting the conclusions of this article will be made available by the authors, without undue reservation.

## Ethics Statement

The studies involving human participants were reviewed and approved by The Social Research Ethics Subcommittee, Maynooth University (2407411). The patients/participants provided their written informed consent to participate in this study.

## Author Contributions

KO’S conceived the idea, secured ethical approval, and designed the study. SC wrote and edited overall document. NB collected, cleaned and analysed data. LB and NR completed literature review and first draft of document. NJ collected data. HF edited document and analysed data. All authors contributed to the article and approved the submitted version.

## Funding

This research was funded by the SFI/EI/IDA COVID-19 Rapid Response (Award ID: 20/COV/0151).

## Conflict of Interest

The authors declare that the research was conducted in the absence of any commercial or financial relationships that could be construed as a potential conflict of interest.

## Publisher’s Note

All claims expressed in this article are solely those of the authors and do not necessarily represent those of their affiliated organizations, or those of the publisher, the editors and the reviewers. Any product that may be evaluated in this article, or claim that may be made by its manufacturer, is not guaranteed or endorsed by the publisher.
